# Pulmonale Mitbeteiligung bei seltenen systemischen Erkrankungen – Teil 1: Pulmonale Manifestationen im Kontext hämatologischer, immundefizitärer, histiozytärer und hepatischer Grunderkrankungen sowie chronisch-entzündlicher Darmerkrankungen

**DOI:** 10.1007/s00117-025-01497-z

**Published:** 2025-09-04

**Authors:** Jasmin Happe, Thomas Frauenfelder

**Affiliations:** Institut für Diagnostische und Interventionelle Radiologie, Universitätsspital Zürich, Rämistrasse 100, 8091 Zürich, Schweiz

**Keywords:** Pulmonale Manifestation, Hämatologische Neoplasien, Immundefizienz, Hepatopathie, Histiozytosen, Pulmonary manifestation, Hematological neoplasms, Immunodeficiency, Liver diseases, Histiocytosis

## Abstract

**Hintergrund:**

Pulmonale Manifestationen treten bei einer Vielzahl multisystemischer Erkrankungen auf und können das Lungenparenchym, die Atemwege, das Gefäßsystem sowie die Atemmuskulatur auf unterschiedliche Weise betreffen. Besonders bei systemischen Grunderkrankungen wie immunologischen und hämatologischen Erkrankungen sind schwere, teils lebensbedrohliche pulmonale Komplikationen gefürchtet. Die in den letzten Jahren gestiegene Inzidenz hämatologischer, gastrointestinaler und hepatologischer Erkrankungen – bedingt nicht zuletzt durch Lebensstilfaktoren in westlichen Industrienationen – unterstreicht die zunehmende klinische Relevanz dieser Entitäten und die Notwendigkeit einer differenzierten Kenntnis ihrer pulmonalen Manifestationsformen. Zusätzliche diagnostische und therapeutische Herausforderungen ergeben sich durch medikamenteninduzierte Lungenschäden sowie durch therapeutische Spätkomplikationen wie etwa nach Stammzelltransplantation.

**Ergebnisse:**

Der hochauflösenden Computertomographie (HRCT) kommt hierbei eine zentrale Rolle zu. Sie ermöglicht die frühzeitige Detektion charakteristischer pulmonaler Veränderungen und bildet damit die Grundlage für eine rechtzeitige therapeutische Intervention.

**Schlussfolgerung:**

Die gezielte HRCT-Diagnostik leistet somit einen entscheidenden Beitrag zur Verbesserung des klinischen Outcomes und zum Erhalt der Lebensqualität betroffener Patienten.

## Abstract (en)

**Background:**

Pulmonary manifestations occur in a wide range of multisystemic diseases and can affect the lung parenchyma, airways, vascular system, and respiratory musculature in various ways. In particular, severe and potentially life-threatening pulmonary complications are of major concern in systemic conditions such as immunological and hematological disorders. The increasing incidence of hematological, gastrointestinal, and hepatological diseases in recent years—driven in part by lifestyle factors in Western industrialized nations—highlights the growing clinical relevance of these entities and the need for a comprehensive understanding of their pulmonary manifestations. Additional diagnostic and therapeutic challenges arise from drug-induced lung injury and late complications of therapy, such as those following stem cell transplantation.

**Results:**

High-resolution computed tomography (HRCT) plays a pivotal role in this context. It allows for the early detection of characteristic pulmonary abnormalities and thus forms the basis for timely therapeutic intervention.

**Conclusion:**

Targeted HRCT imaging is therefore crucial to improving clinical outcomes and preserving the quality of life in affected patients.

Pulmonale Manifestationen finden sich bei zahlreichen multisystemischen Erkrankungen (Abb. 1), wobei Lungenparenchym, Atemwege, Gefäßsystem, Pleura sowie die Atemmuskulatur in unterschiedlichster Weise betroffen sein können [1]. Dabei kann die pulmonale Beteiligung entweder als primäre Manifestation der zugrunde liegenden Multisystemerkrankung, als sekundäre Komplikation oder als Folge therapeutischer Maßnahmen auftreten [2].

**Abb. 1:**
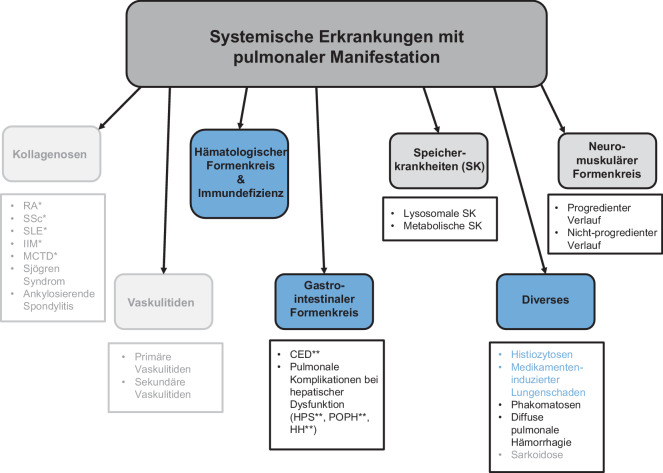
Systemische Erkrankungen mit pulmonaler Manifestation. *RA rheumatoide Arthritis, *SSc* systemische Sklerose, *SLE* systemischer Lupus erythematodes, *IIM* idiopathische inflammatorische Myositiden, *MCTD* Mischkollagenosen **CED Chronisch-entzündliche Darmerkrankungen, *HPS* hepatopulmonale Syndrome, *POPH* portopulmonale Hypertension, *HH* hepatischer Hydrothorax. Die *blau* gefärbten Bereiche werden hier, in Teil 1 dieses Artikels behandelt. Die *grau* hinterlegten Felder sind Gegenstand von Teil 2 dieses Artikels [3]. Die *abgeblassten* Bereiche wurden in vier weiteren Beiträgen dieser Ausgabe [4–7] bereits ausführlich abgehandelt, gehören jedoch ebenfalls zum Formenkreis *systemischer Grunderkrankungen mit pulmonaler Manifestation* und sind hier der Vollständigkeit halber mit aufgeführt

Die hochauflösende Computertomographie (HRCT) ist ein zentrales Instrument zur Diagnose und Verlaufskontrolle pleuropulmonaler Veränderungen [2]. Sie ist sensitiver als das konventionelle Röntgenbild und ermöglicht eine frühzeitige Erkennung pathologischer Befunde [8]. Lungenfunktionsprüfungen wie forcierte Vitalkapazität (FVC) und Diffusionskapazität der Lunge für Kohlenmonoxid (DLCO) sind zwar klinischer Standard, jedoch durch breite Normalbereiche und Messabweichungen limitiert [9]. In solchen Fällen liefert die HRCT wertvolle Zusatzinformationen.

Zudem hilft die HRCT, invasive Verfahren wie Biopsien zu vermeiden, die bei reduziertem Allgemeinzustand oft nur eingeschränkt möglich sind [10–12], und unterstützt durch ihre hohe räumliche Auflösung gezielt die Biopsieplanung – etwa zur Abgrenzung entzündlicher von fibrotischen Arealen [2]. Auch für Verlaufskontrolle und Therapie-Monitoring ist die HRCT unverzichtbar [2], da sie aufgrund der exzellenten räumlichen Auflösung in der Lage ist, akut-entzündliche Alveolitisbereiche von postentzündlich-fibrotischen Prozessen zu differenzieren.

Moderne Bildgebung hat sich zudem als unverzichtbar im klinischen Alltag etabliert, insbesondere für die Verlaufsbeurteilung und das Therapie-Monitoring [2]. Ein dominierendes Ground-Glass-Muster kann auf reversible alveolär-entzündliche Infiltrate hinweisen und ist bei frühzeitiger Therapie mit einem besseren Ansprechen assoziiert [9].

Gerade bei multisystemischen Erkrankungen mit pulmonaler Beteiligung, die Morbidität und Mortalität wesentlich beeinflusst, spielt die HRCT – kombiniert mit Klinik und Labor – eine zentrale Rolle für die frühzeitige Erkennung, präzise Charakterisierung, Quantifizierung und Verlaufsbeurteilung.

Im folgenden Abschnitt „Teil 1“ liegt der Schwerpunkt auf immunologischen und hämatologischen Krankheitsbildern sowie chronisch-entzündlichen Erkrankungen mit primär abdominaler Manifestation. Angesichts der bei diesen Erkrankungen häufig erforderlichen, komplexen medikamentösen Therapieschemata, welche mit teils schwerwiegenden Nebenwirkungsprofilen einhergehen, wird zudem ein besonderer Fokus auf die pulmonale Beteiligung im Rahmen medikamenteninduzierter Lungenschäden gelegt.

## Hämatologische Erkrankungen

**Abb. 2:**
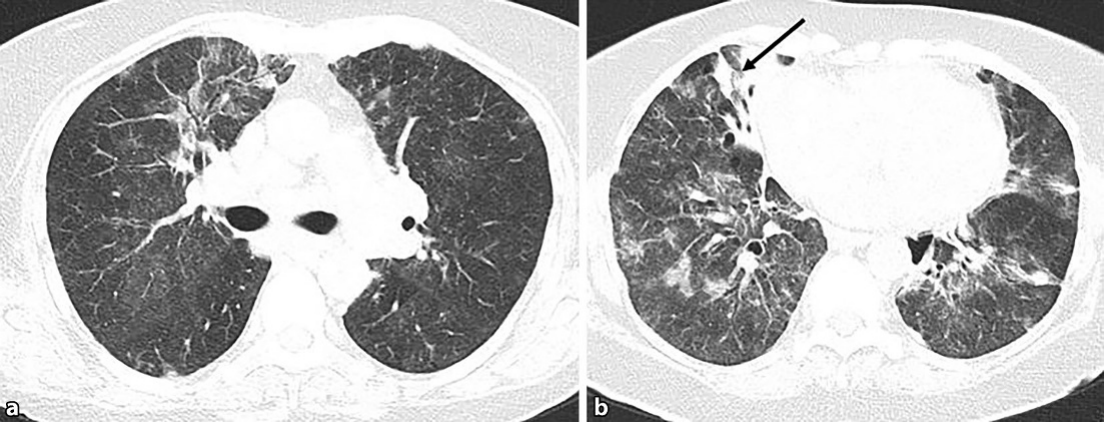
B‑Zell-Lymphom. CT Thorax einer 70-jährigen Patientin mit Abbildung des axialen Lungenfensters auf Höhe der Oberlappen (**a**) sowie auf Höhe der Unterlappen (**b**): ubiquitär-fleckige, primär peribronchial verteilte Konsolidationen sowie Ground-Glass-Opazitäten mit Aerobronchogrammen. Zusätzlich flächige Konsolidation im medialen Mittellappen rechts (*schwarzer Pfeil*). Begleitend zeigen sich diffuse interstitielle Verdickungen. Radiologisch ist das klassische Bild eines pulmonalen Lymphombefalls zu erkennen, was histologisch bestätigt werden konnte. Begleitend ausgedehnte hilomediastinale und axilläre Lymphadenopathie (nicht abgebildet)

Hämatologische Erkrankungen, insbesondere Lymphome, können sich in der Lunge sowohl primär als auch sekundär manifestieren. Primäre pulmonale Lymphome sind selten und meist vom Non-Hodgkin-Typ. In diesen Fällen kann der pulmonale Lymphombefall mit mediastinaler Lymphadenopathie einhergehen, jedoch ohne Hinweise auf eine extrathorakale Ausbreitung innerhalb von mindestens 3 Monaten nach der Erstdiagnose. Zu den häufigsten primären Lymphomen gehören niedriggradige B‑Zell-Lymphome (MALT-Lymphome) und hochgradige B‑Zell-Lymphome [13]. Sekundäre pulmonale Lymphome treten häufiger auf und umfassen Hodgkin- und Non-Hodgkin-Lymphome (Abb. 2).

Im Rahmen hämatologischer Neoplasien kann eine Lungenbeteiligung jedoch nicht nur als maligne Manifestation auftreten, sondern auch die Entwicklung sekundärer, primär benigner pulmonaler Begleiterkrankungen ist möglich. Ein Beispiel dafür ist die Assoziation zwischen verschiedenen hämatologischen Malignomen und einer erworbenen Form der pulmonalen Alveolarproteinose (PAP) [1]. Wie bei der primären PAP kommt es charakteristischerweise zu einer intraalveolären Akkumulation von lipoproteinreichem Material, was zu einem geografischen Crazy-Paving-Pattern führt.

Darüber hinaus können auch andere hämatologisch-systemische Erkrankungen, wie etwa das akute Thoraxsyndrom bei Sichelzellanämie [1], mit spezifischen pulmonalen Komplikationen einhergehen. Infolge der Hämoglobinopathie kommt es hierbei zu vasookklusiven Ereignissen in der pulmonalen Gefäßversorgung mit möglicher Ausbildung von parenchymalen Infarktarealen. Chronische Gefäßverschlüsse erhöhen im weiteren Verlauf das Risiko für die Entwicklung einer pulmonalen Hypertonie [1].

Ein weiterer pulmonaler Manifestationsweg zeigt sich zudem nicht selten im Zusammenhang mit Stammzelltransplantationen [14]. Frühkomplikationen (< 120 Tage) können sowohl infektiöser (insbesondere bedingt durch opportunistische Erreger) als auch nichtinfektiöser Genese sein, wie etwa Lungenödeme, das idiopathische Pneumoniesyndrom basierend auf einem diffusen Lungenschaden oder die medikamentenassoziierte Pneumonitis [15]. Zu den häufigsten opportunistischen Pathogenen zählen in der Frühphase nach Transplantation die Cytomegaloviren sowie Aspergillus-Spezies [16]. Leung et al. [16] konnten in diesem Zusammenhang zeigen, dass in den ersten 30 Tagen nach Transplantation pilzbedingte pulmonale Infektionen dominieren (82 % der Fälle), während im Zeitraum zwischen Tag 31 und 100 vor allem virale Erreger mit 62 % der Fälle in den Vordergrund treten. Die Graft-versus-Host-Reaktion (GvHD) stellt zudem eine bedeutende Spätkomplikation der Stammzelltransplantation dar. Diese manifestiert sich histopathologisch vor allem als Bronchiolitis obliterans [1], seltener als organisierende Pneumonie oder Lungenfibrose („non-classifiable interstitial pneumonia“).

Darüber hinaus stellen transfusionsassoziierte Lungenkomplikationen einen besonders zu berücksichtigen Aspekt im Formenkreis der hämatologischen Erkrankungen dar. Nach Verabreichung von Blutprodukten kann es zur Entwicklung eines transfusionsassoziierten Lungenversagens (TRALI) kommen, typischerweise während oder innerhalb von 6 Stunden nach der Transfusion. Bildmorphologisch zeigen sich interlobuläre septale Verdickungen, fleckige Ground-Glass-Opazitäten (GGO) und/oder Konsolidierungen [17]. In schweren Fällen kann sich ein diffuses GGO-Pattern ausbilden. Pleuraergüsse können bei TRALI zwar auftreten, sind jedoch kein obligater Befund. Die gleichzeitige Präsenz von pulmonalen Stauungszeichen, Pleuraergüssen sowie Hinweisen auf einen erhöhten Rechtsherzdruck spricht hingegen eher für eine transfusionsassoziierte Volumenüberladung (TACO) [17].

## Immundefizienzerkrankungen

Immundefizienzkrankheiten stellen eine heterogene Gruppe von Erkrankungen dar, die durch eine pathologisch erhöhte Anfälligkeit für Infektionen sowie bestimmte maligne Erkrankungen gekennzeichnet sind. Sie lassen sich grundsätzlich in primäre (angeborene) und sekundäre (erworbene) Immundefizienzen unterteilen.

*Primäre Immundefizienzen (PID)* beruhen auf genetisch bedingten Defekten des Immunsystems und weisen eine zunehmende Heterogenität auf. Die Zahl neu identifizierter genetischer Veränderungen wächst stetig. Mit einem Anteil von etwa 70–75 % stellen Antikörpermangelsyndrome die häufigste Subgruppe der PID dar [18]. Respiratorische Manifestationen betreffen sowohl die oberen als auch die unteren Atemwege. Besonders Komplikationen der unteren Atemwege gelten als klinisch relevant, da sie zu strukturell irreversiblen Schäden des Lungenparenchyms führen können. Dies beeinträchtigt die Lungenfunktion erheblich und wirkt sich negativ auf die Prognose aus [18]. Die respiratorischen Veränderungen lassen sich grundsätzlich in infektiöse und nichtinfektiöse Manifestationen einteilen. In den ersten Krankheitsjahren dominieren Infektionen als häufigste klinisch-symptomatische Präsentationsform. Typischerweise äußern sie sich in rezidivierenden und langanhaltenden Infektionen der oberen Atemwege (z. B. Rhinosinusitis, Otitis media) und der unteren Atemwege (z. B. Bronchitis, Bronchiektasen, Pneumonien). Bei Patienten mit Antikörpermangelsyndromen werden in frühen Krankheitsphasen Exazerbationen vor allem durch *Haemophilus influenzae* und *Streptococcus pneumoniae* verursacht. Im Verlauf der Erkrankung, mit zunehmender Lungenschädigung, dominieren zunehmend *Pseudomonas aeruginosa* und *Staphylococcus aureus* das Erregerspektrum [19]. Im Vergleich zu immunkompetenten Patienten verlaufen respiratorische Infektionen bei PID-Patienten schwerer, sind langwieriger und werden häufiger durch atypische oder opportunistische Erreger verursacht. Dies zeigt sich typischerweise anhand der S.P.U.R.-Kriterien: *schwer, persistierend, ungewöhnlich, rezidivierend*. Unkontrollierte Infektionen können schwerwiegende pulmonale Komplikationen wie Lungenabszesse, Empyeme oder Pneumatozelen zur Folge haben [18]. Im weiteren Krankheitsverlauf führen rezidivierende Infektionen dann zunehmend zu chronisch-destruktiven Veränderungen. Diese manifestieren sich entweder als strukturelle Schädigungen der Atemwege, wie etwa in Form von Bronchiektasen, Bronchialwandverdickungen und Air-Trapping, oder des Lungenparenchyms, etwa durch pulmonale Granulombildungen oder die Entwicklung interstitieller Lungenerkrankungen einschließlich postentzündlicher, narbig-fibrotischer Parenchymveränderungen. Diese sekundären, nichtinfektiösen, strukturellen Schädigungen erhöhen jedoch wiederum das Risiko für infektiöse Manifestationen.

**Abb. 3:**
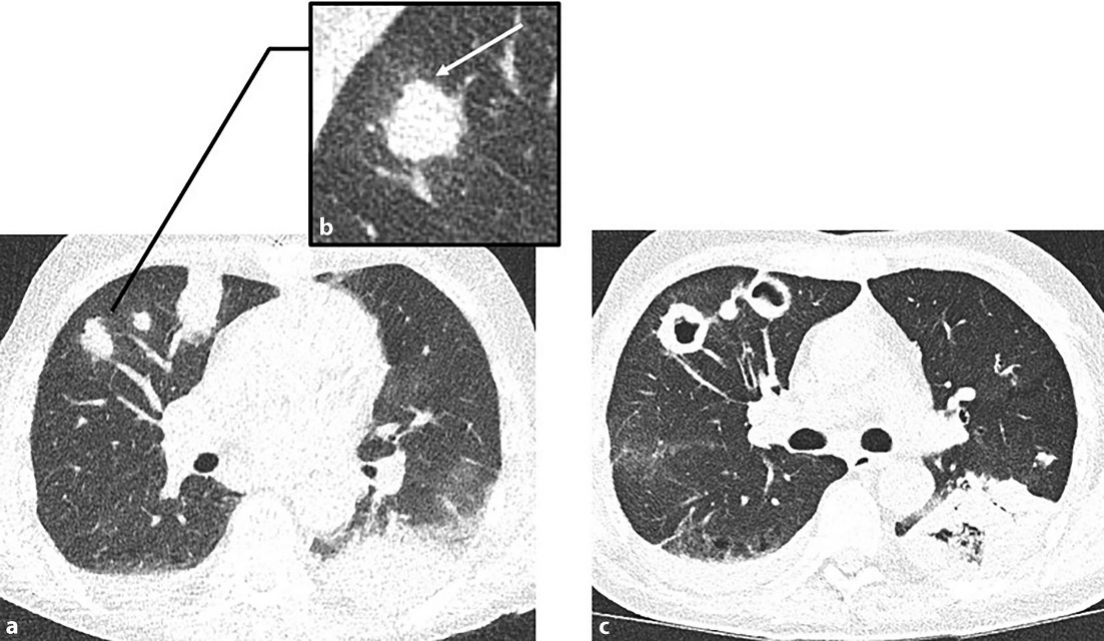
Angioinvasive Aspergillose. 60-jähriger immunsupprimierter Patient unter Hochdosis-Kortikosteroidtherapie bei Status nach Organtransplantation. Eine Hochdosis-Kortikosteroidtherapie stellt einen Risikofaktor für die Entwicklung einer angioinvasiven Aspergillose dar. **a** Axiale CT-Thorax im Lungenfenster: multiple pulmonale, massenartige Läsionen sowie eine Konsolidation im linken Unterlappen, was mit einer angioinvasiven vaskulären Okklusion durch Aspergillus-Hyphen und den daraus resultierenden pulmonalen Infarkten vereinbar ist. In der Vergrößerungsaufnahme in **b** ist zudem das klassische „Halo sign“ sichtbar, das histopathologisch hämorrhagischen Infarkten entspricht. **c** Verlaufskontrolle nach 3 Wochen, bei der eine zunehmende Kavitation der ursprünglichen Konsolidationen erkennbar ist. Dies repräsentiert den klassischen zeitlichen Verlauf einer *abheilenden* angioinvasiven Aspergillose, bei welcher eine Separation des zentral-nekrotischen vom noch vitalen Lungengewebe stattfindet, was zur Ausbildung von Kavitationen führt

Auch *sekundäre Immundefizienzen* gehen mit einer erhöhten Anfälligkeit für Infektionen einher, die – abhängig vom zugrunde liegenden Immundefekt – schwerer, rascher progredient und häufig durch ein spezifisches Erregerspektrum gekennzeichnet sind [20]. Zu den häufigsten Ursachen zählen hämatologische Neoplasien (z. B. Leukämien, Lymphome), HIV-Infektionen sowie medikamenteninduzierte Immunsuppression, etwa im Rahmen von Organtransplantationen oder Autoimmunerkrankungen. Patienten unter immunsuppressiver Therapie oder mit HIV-Infektion sind besonders gefährdet für Infektionen mit opportunistischen Erregern. Zu den häufigsten zählen Pilze wie Pneumocystis jirovecii, Cryptococcus neoformans und Aspergillus-Arten (Abb. 3), opportunistische Viren wie das Cytomegalovirus und das Herpes-simplex-Virus sowie spezielle bakterielle Erreger wie Nocardia-Arten und Methicillin-resistenter *Staphylococcus aureus* (MRSA; [20, 21]).

Sowohl bei primären als auch bei sekundären Immundefizienzen ist zudem das Risiko für lymphoproliferative Erkrankungen erhöht. Dies umfasst sowohl benigne Veränderungen wie die pulmonale noduläre lymphoide Hyperplasie, follikuläre Bronchiolitis und lymphoide interstitielle Pneumonie als auch maligne Entitäten, insbesondere Leukämien und Lymphome [18]. Hinsichtlich einer allgemein erhöhten Inzidenz solider Tumoren ist die Datenlage eher uneinig. Die aktuelle Studienlage deutet jedoch darauf hin, dass tendenziell nur spezifische Tumorarten aufgrund der eingeschränkten Funktion des Immunsystems von einer erhöhten Krebsinzidenz betroffen sind, anstatt des ursprünglich angenommenen ubiquitär erhöhten Malignomrisikos [22].

Eine besondere Rolle in dem pathogenen Formenkreis der Immundefizienzerkrankungen nimmt das humane Immundefizienz-Virus (HIV) ein. Dieses führt durch die gezielte Infektion von Zellen des Immunsystems, bevorzugt der CD4^+^-T-Helferzellen, zu einer pathologischen Erniedrigung des CD4/CD8-Verhältnisses mit einer folglich ausgeprägten Störung der Immunhomöostase [1]. In der Folge resultiert eine charakteristische Anfälligkeit für opportunistische Erreger, insbesondere bei CD4-Zellzahlen unter 200/μl [1]. Neben dieser erhöhten Infektionsanfälligkeit ist auch die Inzidenz bestimmter maligner Erkrankungen gesteigert, etwa des Kaposi-Sarkoms oder des bronchogenen Karzinoms [1]. Zusätzlich besteht eine erhöhte Prädisposition für nichtinfektiöse pulmonale Erkrankungen, insbesondere chronisch-obstruktive Lungenerkrankungen (COPD). Ebenfalls beschrieben ist ein gesteigertes Risiko für die Entwicklung einer HIV-assoziierten pulmonal-arteriellen Hypertonie, wahrscheinlich infolge eines virusvermittelten vaskulären Remodelings [1].

Darüber hinaus kann eine Immundefizienz ebenfalls die Entwicklung einer sekundären pulmonalen Alveolarproteinose begünstigen [23]. Hierbei beeinträchtigt die gestörte Immunfunktion die effektive Clearance von Surfactant sowie anderen extrazellulären Bestandteilen aus dem Alveolarraum, was zu einer pathologischen Akkumulation dieser Substanzen in den Alveolen führt.

## Histiozytäre Erkrankungen

Histiozytosen sind seltene Erkrankungen, bei denen es zu einer pathologischen Akkumulation von Makrophagen, dendritischen Zellen oder Monozyten-abgeleiteten Zellen in verschiedenen Geweben und Organen kommt. Die überarbeitete Klassifikation histiozytärer Erkrankungen aus dem Jahr 2016 [24] unterteilt diese in 5 Gruppen. Ein wesentlicher Unterschied zur alten Klassifikation ist die Neubewertung der Unterscheidung zwischen Langerhans- (LCH) und Nicht-Langerhans-Histiozytosen, da etwa 20 % der Patienten mit der Erdheim-Chester-Erkrankung (ECD) auch LCH-Läsionen aufweisen. Deshalb wurden LCH und ECD in der neuen Klassifikation in einer gemeinsamen Gruppe zusammengefasst [24].

Innerhalb der *Langerhans-Zell-Histiozytose (LCH)* zeigt sich ein breites klinisches Spektrum, das von *spontan selbstlimitierenden* Verläufen bis hin zu *fulminant-progredienten* Formen reicht. Die Erkrankung kann sich entweder:

- als multisystemische Organerkrankung manifestieren – auch bekannt als Letterer-Siwe-Erkrankung, die überwiegend im Kindesalter auftritt und mit einer ungünstigen Prognose einhergeht (eine pulmonale Beteiligung liegt in etwa 15 % der Fälle vor [24]),
- oder als solitäre Organmanifestation, die vorwiegend junge Erwachsene betrifft und typischerweise Knochen, zentrales Nervensystem (ZNS) oder Lunge involviert [25].

Im Falle einer pulmonalen Manifestation im Erwachsenenalter zeigt sich eine starke Assoziation der Erkrankung mit dem Rauchen. Zudem wurde eine Assoziation der LCH mit anderen hämatologischen Neoplasien, insbesondere akuten Leukämien, berichtet [26].

Histopathologisch zeigt sich eine Proliferation der Langerhans-Zellen im Epithel der Bronchiolen mit folglicher Ausbildung von Granulomen. Es wird postuliert, dass die Entwicklung der zellulären Granulome mit einer umgebenden Fibrose einhergeht, die in einer Traktion auf die zentralen Bronchiolen und damit in einer zystenartigen Deformation der Atemwege mündet [27].

**Abb. 4:**
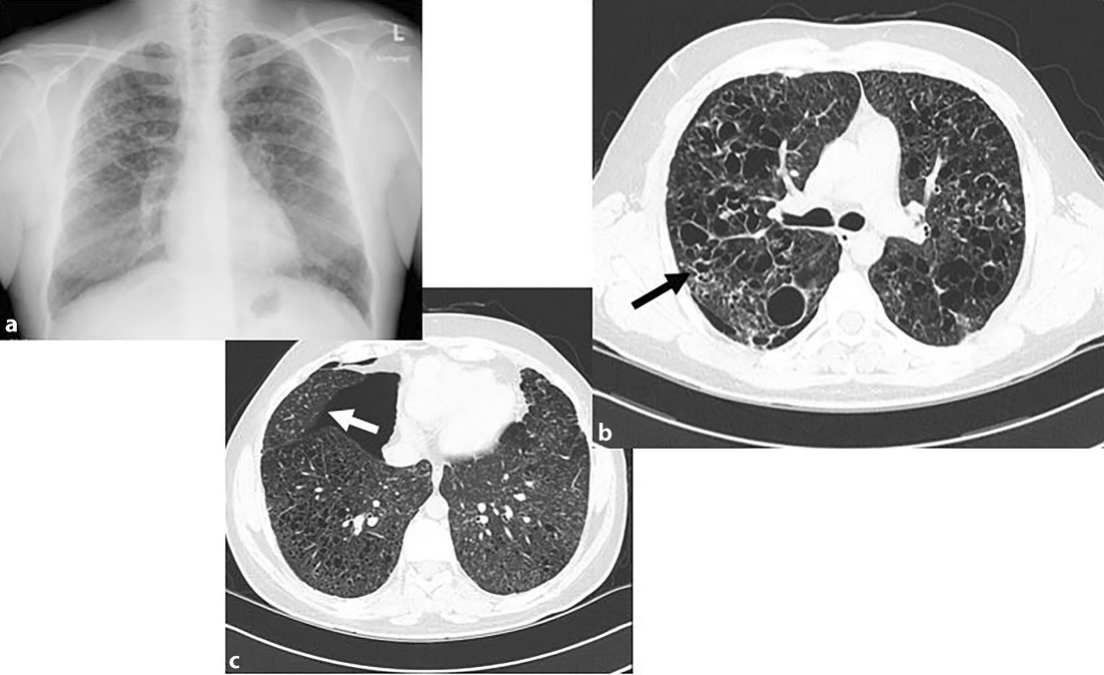
Typisches Bild einer Langerhans-Zell-Histiozytose (LZH). **a** Posterior-anteriore Röntgenaufnahme mit dem charakteristischen apikobasal betonten Gradienten der zystischen Lungenerkrankung, welche sich im Röntgenbild häufig vorwiegend durch ein führendes retikuläres Muster manifestiert. Die ergänzende CT-Thorax (**b**) desselben Patienten auf Höhe der Lungenoberlappen zeigt multiple, teils bizarr konfigurierte sowie teils irregulär-wandverdickte Lungenzysten (*schwarzer Pfeil*). Das CT-Verlaufsbild (**c**) illustriert eine typische Komplikation – die Ausbildung eines Pneumothorax, hier mit einer ausgedehnten Pneumothoraxlamelle rechts (*weißer Pfeil*)

Diese Pathophysiologie spiegelt sich auch in der bildgebenden Diagnostik wider:

- Im Frühstadium dominieren peribronchiale, irreguläre Noduli (< 10 mm), mit Verteilungspräferenz in den Ober- und Mittellappen bei charakteristischer Aussparung der kostophrenischen Winkel.
- Im weiteren Verlauf kommt es zur Kavitation der Noduli, die sich zu dickwandigen, bizarr konfigurierten Zysten entwickeln (Abb. 4) und schließlich – im Spätstadium – in dünnwandige Lungenzysten übergehen.
- Im Endstadium der Erkrankung tritt zunehmend eine retikuläre Musterbildung mit fibrotischen Veränderungen auf. In schweren Fällen kann es zum Honeycombing kommen. Die Lungenvolumina bleiben – abgesehen von einem fortgeschrittenen fibrotischen Stadium – meist normal oder erhöht [25].

Komplikationen sind spontane Pneumothoraces durch Ruptur der Lungenzysten [28] sowie eine pulmonale Hypertension.

**Abb. 5:**
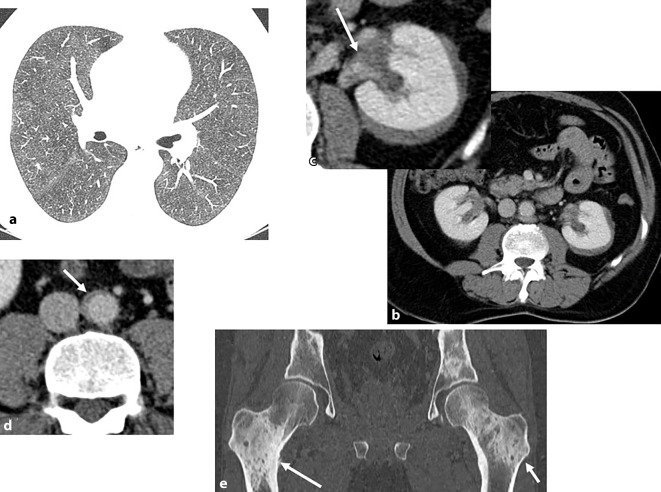
54-jähriger Patient mit bekannter Erdheim-Chester-Erkrankung. **a** Axiales Lungenfenster mit diskretem, jedoch ubiquitär vorherrschendem Ground-Glass. **b** Portalvenöse CT-Aufnahme des Abdomens mit charakteristischer renaler Beteiligung in Form einer ausgedehnten bilateralen, irregulären Infiltration des perirenalen Fettgewebes (Hairy-kidney-Zeichen) und fehlender kortikomedullärer Differenzierung (Featureless-kidney-Zeichen). Zusätzlich retroperitoneale Beteiligung mit periureteralem Weichteilplus um den rechten proximalen Ureter (Vergrößerungsaufnahme **c** , weißer *Pfeil*). **d** Periaortale Infiltration (Coated-aorta-Zeichen) durch Nicht-Langerhans-Zell-Histiozyten, was sich als periaortales, plaqueartiges, hypodenses Weichteilplus um die infrarenale Aorta darstellt (*weißer* *Pfeil*). **e** Koronale Reformatierung im Knochenfenster mit dem charakteristischen Bild einer bilateralen Mehrsklerose metadiaphysär, in diesem Fall führend im Bereich der pertrochantären Region des Femurs beidseits (*weiße Pfeile*), mit begleitend assoziierter kortikaler Verdickung

Die Differenzierung zwischen einer pulmonalen Manifestation der Langerhans-Zell-Histiozytose (LCH) und der *Erdheim-Chester-Erkrankung (ECD) *kann radiologisch herausfordernd sein. Bei der ECD manifestiert sich ein überwiegend retikulär-interstitieller Prozess, der durch die interstitielle Akkumulation von histiozytären Zellen und assoziierten fibrotischen Veränderungen geprägt ist [29]. Diese fibrotischen Veränderungen weisen typischerweise ein subpleural-dominierendes Verteilungsmuster auf, welches differenzialdiagnostisch als Abgrenzung zum primär peribronchialen Verteilungsmuster der LCH hilfreich sein kann [29]. Im Rahmen der interstitiellen Infiltration zeigt sich darüber hinaus bei der ECD eine interlobulär-septale und fissurale Verdickung, die primär ein Smooth-like-Erscheinungsbild hat, während bei der LCH ein nodulärer Charakter überwiegt [29]. Zentrilobuläre noduläre Opazitäten können bei der ECD zwar auftreten, sind jedoch im Vergleich zur LCH weniger ausgeprägt und nicht das dominierende Pattern [29, 30]. Ein weiteres typisches Merkmal der ECD ist die Assoziation mit dem Vorhandensein von Pleuraergüssen und pleuralen Verdickungen, welche bei der LCH ebenfalls selten zu finden sind [29]. Zudem sind bei der ECD diffuse multifokale Ground-Glass-Opazitäten (Abb. 5) sowie perikardiale Verdickungen und Flüssigkeitsansammlungen charakteristisch. Extrapulmonale Manifestationen, wie perivaskuläre Infiltrationen im Bereich der Aorta (Abb. 5) sowie auch der Pulmonalarterien oder der Vena cava superior mit assoziiertem perivaskulärem Weichteilplus, sind ebenfalls bei der ECD vergleichsweise häufig anzutreffen [28, 30].

Im Gegensatz zur LCH und der ECD ist die *Rosai-Dorfman-Erkrankung (RDD)* primär mit einer Beteiligung der Lymphknoten assoziiert. In seltenen Fällen kann jedoch auch eine pulmonale Manifestation auftreten. Hierbei zeigen sich in der HRCT noduläre Opazitäten, interstitiell-perilymphatische Verdickungen sowie eine assoziierte Pleuraaffektion [28]. Zudem kann es thorakal zu einer Manifestation im Bereich der mediastinalen Lymphknoten im Sinne einer Lymphadenopathie kommen.

## Gastrointestinale Erkrankungen

### Chronisch-entzündliche Darmerkrankungen

Morbus Crohn und Colitis ulcerosa sind multisystemische Erkrankungen, die sich durch eine Vielzahl extraintestinaler Manifestationen auszeichnen können. Die genaue Pathogenese einer pulmonalen Manifestation ist bislang nicht vollständig geklärt, jedoch deuten aktuelle Forschungsergebnisse auf eine komplexe Interaktion zwischen Darm und Lunge hin – oftmals beschrieben als „gut-lung axis“ [31].

Die pulmonale Beteiligung lässt sich grob in 3 Hauptgruppen unterteilen, wobei suppurative Bronchiektasen die häufigste Manifestationsform darstellen [1]:

- *Erkrankungen der großen Atemwege:* Hierzu zählen Bronchiektasen, Tracheobronchitis (im Falle einer chronischen Inflammation kann dies zu Stenosen oder bindegewebsartigen „webs“ führen), Glottis- bzw. subglottische Stenosen sowie chronisch oder eitrig verlaufende Bronchitiden [2, 31].
- *Erkrankungen der kleinen Atemwege:* Hier kommt es insbesondere zum Auftreten einer Bronchiolitis obliterans, einer granulomatösen Bronchiolitis oder diffusen Panbronchiolitis [31].
- *Parenchymatöse Beteiligung:* Am häufigsten findet sich hier das Pattern einer organisierenden Pneumonie (OP), beschrieben sind jedoch auch die nichtspezifische interstitielle Pneumonie (NSIP), die gewöhnliche interstitielle Pneumonie (UIP), die akute interstitielle Pneumonie (AIP), eosinophile Pneumonie sowie das Vorhandensein nekrobiotischer Knötchen [31].

Die nekrobiotischen Knötchen können bildmorphologisch septischen Embolien oder einer Granulomatose mit Polyangiitis ähneln und sollten differenzialdiagnostisch berücksichtigt werden [2]. Eine Beteiligung seröser Kompartimente, wie Perikarditis oder Pleuritis, sowie vaskuläre Komplikationen (z. B. venöse Thromboembolien oder Vaskulitiden) sind seltener beschrieben [31].

### Pulmonale Komplikationen bei hepatischer Dysfunktion

Das hepatopulmonale Syndrom (HPS) ist ein klinisches Syndrom, das durch die Trias aus einer Hepatopathie (meist in Form einer Leberzirrhose), einer Dilatation des pulmonalen Gefäßsystems, insbesondere die Lungenperipherie der Unterlappen betreffend, sowie einer gestörten Oxygenierung gekennzeichnet ist [32]. Im Verlauf der hepatischen Grunderkrankung kommt es zu einer pathologischen Erweiterung des pulmonalen Gefäßsystems, insbesondere auf präkapillärer und kapillärer Ebene, aber auch in Form von pulmonalen oder pleuralen arteriovenösen Malformationen (AVM; [1]). Diese vaskulären Veränderungen führen zu einer gestörten Ventilations-Perfusions-Verteilung und zu einem lageabhängigen Rechts-Links-Shunt [1]. Die Pathophysiologie beruht u. a. auf einer Dysregulation der pulmonalen Angiogenese, einer vermehrten Stickstoffmonoxid(NO)-Bildung sowie einer Endotheldysfunktion [33].

**Abb. 6:**
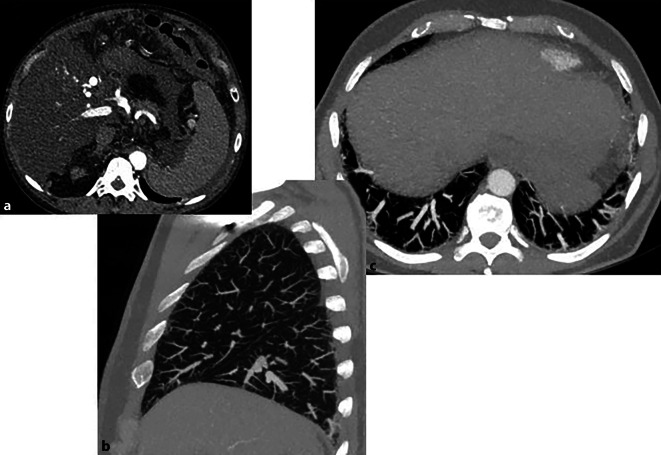
Hepatopulmonales Syndrom bei Leberzirrhose. **a** Axiale CT-Abdomenaufnahme: nodulär-zirrhotischer Umbau des Leberparenchyms sowie eine im Rahmen der portalen Hypertension begleitende Splenomegalie. **b** Axiale CT-Thorax: Zeichen eines hepatopulmonalen Syndroms mit bilateraler Dilatation peripher-subpleuraler Pulmonalarterienäste sowie einer vermehrten Anzahl terminaler Gefäßverzweigungen bis zur Pleura. **c** Sagittale Maximumintensitätsprojektions(MIP)-Rekonstruktion bestätigt die Ausdehnung der pulmonal-arteriellen Gefäßäste bis unmittelbar an die Pleuragrenze

In der CT-Bildgebung zeigen sich eine dilatierte periphere Pulmonalgefäßarchitektur in den basal subpleural gelegenen Lungenabschnitten (Abb. 6), basal-subpleurale Teleangiektasien sowie eine vermehrte Zahl terminaler pleuranaher Gefäßverzweigungen [34].

Demgegenüber ist die portopulmonale Hypertonie (POPH) durch das Auftreten einer pulmonal-arteriellen Hypertonie im Kontext einer portalen Hypertension charakterisiert. Im Gegensatz zum HPS resultiert POPH in einem erhöhten pulmonalvaskulären Widerstand, wodurch sich ein klinisches Bild ergibt, das der idiopathischen pulmonal-arteriellen Hypertonie ähnelt [35]. Zusätzlich kann sich bei Patienten mit Leberzirrhose und ausgeprägtem Aszites ein hepatischer Hydrothorax (HH) entwickeln [35]. Darüber hinaus zeigen Zirrhosepatienten aufgrund einer dysregulierten Immunantwort im Sinne einer „cirrhosis-associated immune dysfunction“ (CAID) eine signifikant erhöhte Anfälligkeit für pulmonale Infektionen [35].

## Medikamenteninduzierte pulmonale Beteiligung

Da Erkrankungen des systemischen Formenkreises häufig mit einem breiten medikamentösen Spektrum behandelt werden, ist auch das Risiko therapeutischer Nebenwirkungen in diesem Patientenkollektiv signifikant erhöht. Speziell zytotoxische Chemotherapeutika sowie Medikamente des rheumatologischen Formenkreises sind mit einem erhöhten Risiko für medikamenteninduzierte Lungenschäden (MIPI) assoziiert [17]. Die Inzidenz medikamenteninduzierter pulmonaler Schädigungen ist in den letzten Jahren signifikant gestiegen, vermutlich bedingt durch die zunehmende Aggressivität therapeutischer Regime, insbesondere im Zusammenhang mit zytotoxischen Chemotherapeutika sowie im rheumatischen Formenkreis, wo das Grundprinzip „hit hard and early“ essenziell ist für die Prognose der Patienten. Die Diagnosestellung gestaltet sich jedoch häufig als herausfordernd, da die Befunde oft unspezifische Veränderungen in der HRCT zeigen.

**Abb. 7:**
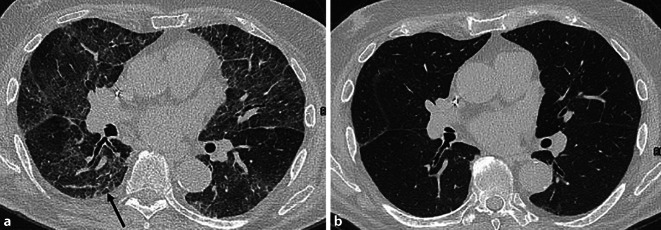
CT-Thorax eines männlichen Patienten mit bekannter rheumatoider Arthritis und kürzlich initiierter Methotrexat-Therapie, der sich notfallmäßig mit progredienter Dyspnoe und Husten vorstellte. **a** Ausgedehnte peribronchovaskulär und subpleural betonte Ground-Glass-Opazitäten, akzentuierte interlobuläre Septen sowie subpleurale Retikulationen (*schwarzer Pfeil*). Das radiologische Muster wurde als Methotrexat-induzierte Pneumonitis im Sinne eines fibrosierenden Musters nichtspezifischen interstitiellen Pneumonie (NSIP) interpretiert, woraufhin die Methotrexat-Therapie umgehend abgesetzt wurde. **b** Kontrolluntersuchung 4 Wochen später: vollständige Rückbildung der pulmonalen Veränderungen – mit sowohl diagnostischem als auch therapeutischem Aussagewert

Die Diagnose erfordert eine sorgfältige Kombination aus Wissen über die häufigsten durch MIPI ausgelösten pulmonalen Manifestationsformen sowie Informationen über die individuell angewendeten Therapieregime. In diesem Zusammenhang haben Sridhar et al. [17] einen diagnostischen Approach entwickelt, der die sechs häufigsten mit MIPI assoziierten radiologischen Patterns umfasst. Das Vorliegen entsprechender radiologischer Befunde in Kombination mit der klinischen und therapeutischen Gesamtkonstellation sollte zumindest differenzialdiagnostisch an eine MIPI denken lassen. Diese 6 Patterns umfassen:

- Sarkoidose-ähnliches Muster
- Zentrilobuläre Ground-Glass-Noduli: Diese Ground-Glass-Noduli korrelieren histopathologisch jedoch – im Gegensatz zur klassischen zellulären Bronchiolitis bei der Hypersensitivitätspneumonie – mit einer bronchozentrischen organisierenden Pneumonie oder follikulären Bronchiolitis.
- Diffuses Ground-Glass-Pattern: Dieses CT-Pattern wird im Fall einer MIPI verursacht durch eine diffuse Hämorrhagie, einen diffusen Alveolarschaden, eine organisierende Pneumonie oder eine schwere akute eosinophile Pneumonie.
- Muster der organisierenden Pneumonie (OP): Dieses Muster wird entweder durch eine OP oder eine chronische eosinophile Pneumonie bedingt.
- Linear-septales Muster: Dieses wird im Rahmen der MIPI durch ein interstitielles pulmonales Ödem, TRALI oder eine akute eosinophile Pneumonie hervorgerufen.
- Fibrotisches NSIP-Muster (Abb. 7)

Jedoch sind diese CT-Patterns – wie bereits erwähnt – sehr unspezifisch und müssen stets im Kontext der gesamten klinischen sowie laborchemischen Befunde betrachtet werden. Wichtige Differenzialdiagnosen für diese Muster sind in Tab. 1 aufgeführt.

**Tab. 1:** Alternative (nicht medikamenteninduzierte) Ursachen eines MIPI-Patterns. (Adaptiert nach [17]).

MIPI-Pattern	Häufige Differenzialdiagnosen	Seltene Differenzialdiagnosen
Sarkoidose-ähnlich	Sarkoidose, Lymphangitis carcinomatosa	Silikose, Berryliose
Zentrilobuläre Ground-Glass-Noduli	Hypersensitivitätspneumonitis, respiratorische Bronchiolitis	Follikuläre Bronchiolitis
Diffuses Ground-Glass	Diffuser (nicht medikamenteninduzierter) Alveolarschaden, hydrostatisches Lungenödem, Pneumonie (z. B. Pneumocystis jirovecii)	OP*, diffuse alveoläre Hämorrhagie
OP* oder CEP*	Pneumonie, Malignität (insb. lymphoproliferative Erkrankungen)	Kryptogene OP*, Radiation-Recall, angioinvasive Fungi, Lungeninfarkt („reverse halo sign“)
Linear-septal	Hydrostatisches Lungenödem	Lymphangitis carcinomatosa
Fibrotisch	NSIP**, UIP**	Fibrosierende Hypersensitivitätspneumonitis

*CEP* chronische eosinophile Pneumonie, *MIPI* medikamenteninduzierte Lungenschäden, *NSIP* nichtspezifische interstitielle Pneumonie, *OP* organisierende Pneumonie, *UIP* gewöhnliche interstitielle Pneumonie

## Fazit

- Zusammenfassend lässt sich festhalten, dass das Spektrum pulmonaler Manifestationen bei systemischen Erkrankungen äußerst vielfältig ist. Es umfasst sowohl unmittelbare pathophysiologische Effekte der Grunderkrankung als auch sekundäre Komplikationen wie etwa infektiöse, vaskuläre oder medikamenteninduzierte Veränderungen.
- Für eine präzise und zeitgerechte Diagnose ist ein interdisziplinärer Ansatz unerlässlich.
- Eine enge Zusammenarbeit zwischen Radiologie, Klinik und Pathologie gewährleistet eine fundierte und umfassende Beurteilung der pulmonalen Manifestationen und ist entscheidend für eine erfolgreiche Diagnosestellung und gezielte Therapie.

## Praxistipps – Teil 1

- Bei persistierenden Konsolidationen im Kontext einer klinisch bekannten Immunsuppression sollte differenzialdiagnostisch stets auch an einen pulmonalen Lymphombefall gedacht werden (hierzu Erläuterung 1).
- Bei persistierend malignitätsverdächtigem Befund trotz initial benigner Histologie sollte eine Rebiopsie erwogen werden (hierzu Erläuterung 2).
- Bei systemischen Erkrankungen sollte im passenden klinisch-radiologischen Kontext stets eine mögliche medikamenteninduzierte Pneumonitis bedacht werden. Ein Therapie-Stopp kann diagnostisch und therapeutisch richtungsweisend sein.

## Ergänzende Erläuterungen

1. Bei persistierenden peribronchialen/subpleuralen Konsolidationen oder Ground-Glass-Opazitäten, die das Bild einer organisierenden Pneumonie imitieren, sollte im Rahmen von Immundefizienzerkrankungen stets auch der pulmonale Lymphombefall differenzialdiagnostisch in Betracht gezogen werden und bei möglichem klinischem Verdacht eine bioptische Sicherung erfolgen.
2. Ein unauffälliger Biopsiebefund schliesst eine Malignität nicht aus. Insbesondere bei deutlicher Immunsuppression und fortbestehendem radiologischem Verdacht auf Malignität sollte demnach eine erneute Biopsie im zeitlichen Intervall erwogen werden.
